# ISG20 stimulates anti-tumor immunity *via* a double-stranded RNA-induced interferon response in ovarian cancer

**DOI:** 10.3389/fimmu.2023.1176103

**Published:** 2023-06-05

**Authors:** Zhigao Chen, Min Yin, Haixue Jia, Qian Chen, Hongbing Zhang

**Affiliations:** ^1^ State Key Laboratory of Common Mechanism Research for Major Diseases, Department of Physiology, Institute of Basic Medical Sciences, Chinese Academy of Medical Sciences and School of Basic Medicine, Peking Union Medical College, Beijing, China; ^2^ Department of Obstetrics and Gynecology, Peking Union Medical College Hospital, Chinese Academy of Medical Sciences and Peking Union Medical College, Beijing, China; ^3^ Tianjin Key Laboratory of Radiation Medicine and Molecular Nuclear Medicine,Institute of Radiation Medicine, Chinese Academy of Medical Sciences and Peking Union Medical College, Tianjin, China; ^4^ Thorgene Co., Ltd., Beijing, China

**Keywords:** ovarian cancer, ISG20, dsRNA, IFN- β, RIG-I

## Abstract

Augmentation of endogenous double-stranded RNA (dsRNA) has become a promising strategy for activating anti-tumor immunity through induction of type I interferon (IFN) in the treatment of ovarian carcinoma. However, the underlying regulatory mechanisms of dsRNA in ovarian carcinoma remain elusive. From The Cancer Genome Atlas (TCGA), we downloaded RNA expression profiles and clinical data of patients with ovarian carcinoma. Using the consensus clustering method, patients can be classified by their expression level of core interferon-stimulated genes (ISGs): IFN signatures high and IFN signatures low. The IFN signatures high group had a good prognosis. Gene set enrichment analysis (GSEA) showed that differentially expressed genes (DEGs) were primarily associated with anti-foreign immune responses. Based on results from protein-protein interaction (PPI) networks and survival analysis, ISG20 was identified as a key gene involved in host anti-tumor immune response. Further, elevated ISG20 expression in ovarian cancer cells led to increased IFN-β production. The elevated interferon improved the immunogenicity of tumor cells and generated chemokines that attract immune cells to infiltrate the area. Upon overexpression of ISG20, endogenous dsRNA accumulated in the cell and stimulated IFN-β production through the Retinoic acid-inducible gene I (RIG-I)-mediated dsRNA sense pathway. The accumulation of dsRNA was associated with the ribonuclease activity of ISG20. This study suggests that targeting ISG20 is a potential immune therapeutic approach to treat ovarian cancer.

## 1 Introduction

Tumor neoantigen vaccines and PD-(L)1 inhibitors are promising immunotherapy approaches for the clinical management of multiple tumor types (1–6). Unfortunately, such immunotherapy approaches may not benefit all patients with cancer, particularly those lacking functional T-cell infiltration (7, 8). Induction of type I interferon (IFN) production by cancer cells can enhance immunotherapy responses by altering innate and adaptive immune functions (9–13). Nevertheless, the regulatory mechanisms underlying the effects of type I IFN in malignancies are yet to be clarified.

Increasing evidence indicates that endogenous double-stranded RNA (dsRNA) accumulation can facilitate type I IFN production (9, 12, 14–16). Endogenous dsRNA mainly derived from mitochondrial transcripts, repetitive nuclear sequences, and endogenous retroviruses (ERVs) (17, 18). DNA demethylation, ablation of histone demethylases, stress-mediated mitochondrial permeabilization, and cleavage by ribonuclease can increase the dsRNA burden in cancerous cells (9, 12, 19, 20). Further, endogenous dsRNA accumulation can trigger melanoma differentiation-associated protein 5 (MDA5) or RIG-I activation, thereby upregulating type I IFN expression (18).

ISG20 is a member of the large family of DEDD 3’-5’ exonucleases (21). Evidence has shown that ISG20 can interfere with the replication of various viruses by degrading viral RNA/DNA (21–29). Interestingly, ISG20 can inhibit the replication of the chikungunya virus (CHIKV) by stimulating type I IFN responses in mouse embryonic fibroblasts rather than by degrading viral RNA (30). However, the mechanism of ISG20 inducing IFN was not revealed, and later studies also failed to confirm the induction of IFN by ISG20 (25).

The present study found that ISG20 overexpression resulted in the degradation of endogenous dsRNA into small dsRNA fragments in ovarian cancer cells, thereby triggering IFN-β production *via* the RIG-I dsRNA sensing pathway. In ovarian cancer, high *ISG20* expression was associated with tumor immunogenicity and increased T cells infiltration. Overall, our findings provide a novel extension to understanding of the mechanism underlying type I IFN regulation in ovarian cancer, thereby providing a rational target for combined immunotherapy.

## 2 Materials and methods

### 2.1 Data acquisition

RNA expression profile and clinical features of ovarian cancer patients were obtained from TCGA and Gene Expression Omnibus database. Ovarian cancer patients with incomplete information regarding clinical characteristics or duplicate entries were excluded. Finally, the total data of 375 patients from the TCGA cohort and 278 patients from the GSE9891 datasets were collected.

### 2.2 Analyses of ovarian cancer subtypes and immune infiltration

We used the “ConsensusClusterPlus” R package to classify patients from the TCGA dataset into two clusters. The analytic tools, CIBERSORTX, MCP counter, and ESTIMATE, were utilized to count the tumor-infiltrating lymphocytes.

### 2.3 Identification of DEGs

DEGs were screened using the “limma” package according to the cutoff criteria of adjusted P < 0.05 and fold changes > 2. The expression difference between IFN signature low and high groups was represented by a volcano plot.

### 2.4 GSEA and PPI

For the DEGs between IFN signature low and high groups, GSEA and PPI were conducted on the website (https://string-db.org/).

### 2.5 Cell culture

The ES2, SKOV3, and HEK293T cancer cell lines were kindly provided by Dr. Jiaxing Yang (Peking Union Medical College Hospital). All the cell lines were maintained in DMEM medium supplemented with 10% fetal bovine serum and 1% penicillin/streptomycin at 37°C with 5% CO2. All the cell lines tested negative for mycoplasma using a Mycoplasma Detection kit (C0301S, Beyotime).

### 2.6 shRNA lentivirus production and infection

HEK293T cells were transfected with shRNA pLKO.1 plasmids, lentivirus packaging plasmid pMD2.G and psPAX2 using Lipofectamine 3000. The medium was collected at 72 h after transfection. A polybrene solution (10μg/ml) was applied to promote the infection of ES2 cells with packaged lentiviral particles. 48 h after transduction, ES2 cells were maintained in DMEM medium containing puromycin (3μg/ml) for selection of the stable knockdown cells over 10 days. The shRNA sequences were listed in **Supplementary Table 1**.

### 2.7 Real-time PCR

Following the manufacturer’s protocol, total RNA was extracted using TRIzol reagent (15596018, Thermo Scientific). 0.1μg of total RNA was used to generate cDNA with the PrimeScript reagent kit (RR037A, TaKaRa). Real-time PCR was performed using Eastep qPCR Master Mix (LS2068, Promega) on a Lepgen-96 Real-time PCR system (LEPU). We conducted all Real-time PCR experiments in triplicate and repeated the experiment with new cDNA preparation. Primer sequences were listed in **Supplementary Table 2**.

### 2.8 Western blot

In the presence of phenylmethylsulphonyl fluoride, the cells were harvested and lysed in RIPA buffer (R0010, Solarbio). The cell lysate was heat-denatured for 8 min at 100°C. Then samples were subjected to SDS–PAGE and transferred to 0.2 µm nitrocellulose membranes (10600001, Amersham Potran). The membranes were blocked with 5% non-fat powdered milk in TBST solution for 30 minutes at room temperature (RT). Then the membranes were incubated with the primary antibodies for 1 hour at 37°C and incubated with secondary antibodies for 1 hour at RT. The membranes were visualized with ECL reagent (C510045, Sangon Biotech). Antibody information is presented in **Supplementary Table 3**.

### 2.9 Immunohistochemistry

Immunohistochemistry was carried out on ovarian cancer microarray (HOvaC070PT01, Shanghai Outdo Biotech Company). Antigen retrieval was performed in pH 9.0 EDTA buffer for 3 minutes when the pressure cooker had reached full pressure. The tissue chips were incubated with primary antibodies at 4°C overnight. The tissue chips were incubated with second antibodies for 40 minutes at 37°C. After washing, the protein signal was developed by DAB staining (ZLI-9018, OriGene).

### 2.10 Immunofluorescence staining

SKOV3 cells were planted on glass coverslips in 24-well plates. Cells were transfected with pcmv-ISG20 plasmids using Lipofectamine 3000. After 24 h, the cells were rinsed twice with PBS, and 0.3mL fixation solution (4% formaldehyde in PBS) was added to the well for 20 minutes at RT. Then the cells were incubated with 0.3mL permeabilization solution (0.5% Triton-X100 in PBS) for 20 minutes at RT. Before the immunostaining, the cells were again rinsed twice with PBS and then blocked with 10% goat serum solution for 30 minutes at RT. The coverslips were then incubated with J2 antibody overnight at 4°C, followed by incubation with second antibody for 30 minutes at 37°C. Coverslips were mounted on a slide with Fluoroshield (F6057, sigma) and analyzed with a Nikon HD25 confocal microscope.

### 2.11 dsRNA dot-blot

The PVDF membrane was pre-wetted with pure methanol for 1 minute, followed by equilibration in TBST for 5 minutes. Then 2.5μl total cellular RNA was spotted on the membrane. The membrane was left to dry to fix the RNA for 1.5 h and incubated with J2 antibody overnight at 4°C. Then the membrane was incubated with goat anti mouse antibody for 1 hour at RT. Dot blot reaction was developed using ECL reagent (C510045, Sangon Biotech).

### 2.12 ELISA

Cell culture medium was harvested and centrifuged. ELISA assays were conducted following the manufacturer’s instructions. The level of IFN-β was measured using the Human IFN-β (RK01630, ABclonal) ELISA Kit.

### 2.13 Statistical analyses

Statistical analyses were performed using GraphPad Prism 8 software, and statistical significance was determined by p < 0.05. For comparisons of tumor infiltrating immune cells, Mann Whitney test was used. For comparing human survival curves, a Log-rank (Mantel-Cox) test was used.

## 3 Results

### 3.1 Patients with IFN signatures high subtype ovarian cancer had favorable prognosis

**Figure 1:**
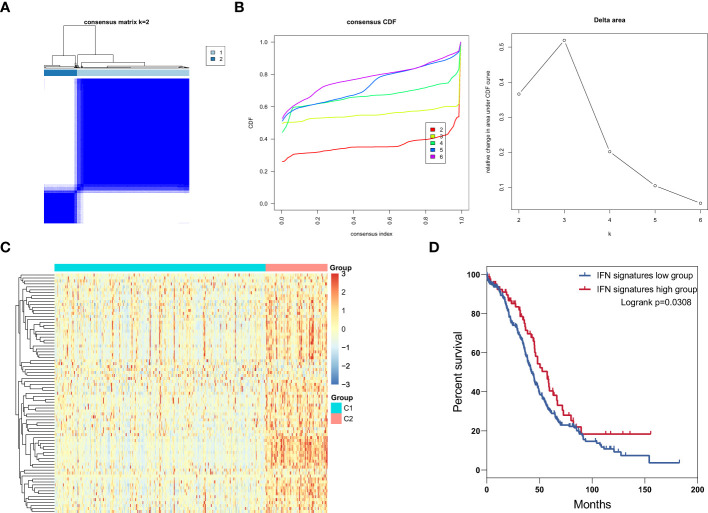
Identification of IFN signature subtypes in ovarian cancer. **(A)** Consensus clustering matrix for k = 2 in patients with ovarian cancer. **(B)** Consensus clustering cumulative distribution function (CDF) and relative change in area under the CDF curve for k = 2 to 6. **(C)** Heatmap of core ISGs expression levels in the low and high IFN signatures group. **(D)** Overall survival curves for patients with ovarian cancer patients in the ISG signature high and low groups.

Type I IFN is critically important in immune system responses to ovarian cancer (9, 31), and triggers the expression of thousands of IFN-stimulated genes (ISGs), which affect antigen presentation, angiogenesis inhibition, protein synthesis modulation, and tumor apoptosis (32); however, mutations in or function-loss of type I IFN signaling pathway-related proteins can interrupt the expression of ISGs (33–35). Categorization of patients based only on their IFN expression level is inappropriate; therefore, we conduct consensus clustering to identify ovarian cancer subtypes based on the expression of core ISGs, which are conserved in evolution and represent the ancestral functions of the IFN system (36). These genes are widely involved in antigen presentation, antiviral responses, IFN suppression, ubiquitination and protein degradation, cell signaling, and apoptosis. In total, 375 patients from the ovarian cancer cohort were classified into two clusters based on expression levels of core ISGs (**Figures 1A, B**), and heatmap analysis showed that cluster 2 presented with high expression of core ISGs (**Figure 1C**). Thus, we categorized patients in cluster 2 as the IFN signatures high group and those in cluster 1 as the IFN signatures low group. Survival analysis illustrated that the IFN signatures high group was associated with favorable clinical outcome (**Figure 1D**).

### 3.2 Identification of *ISG20* as a key gene involved in ovarian cancer immune responses

**Figure 2:**
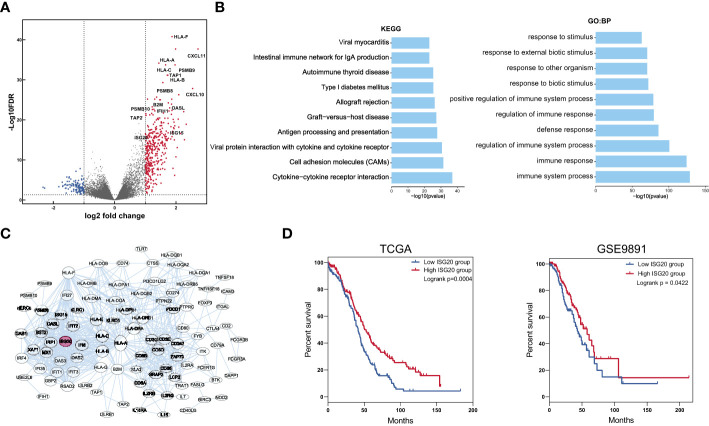
Identification of DEGs and key genes in the IFN signature high and low groups. **(A)** Volcano plot showing DEGs between the low and high IFN signature groups. Cut off values were set at the log_2_ fold-change > 1 or < -1 and the P < 0.05. **(B)** The top 10 biological process and KEGG pathways enriched for DEGs between the low and high IFN signature groups. **(C)** Protein interactions among upregulated DEGs. **(D)** Overall survival curves for patients in the *ISG20* low and high groups in TCGA and GSE9891 datasets. ***P < 0.001 and ****P < 0.0001 by unpaired two-tailed Student’s t-test.

We next identified DEGs and signaling pathways to assess which biological processes were involved in the modulation of patient prognosis. We discovered accumulation of 385 genes that were upregulated in the IFN signatures high group (**Figure 2A**); these upregulated genes were enriched in immune response-related activities, including response to viruses, organisms, and stimuli (**Figure 2B**). These results indicate that anti-foreign immune responses are involved in the modulation of patient prognosis. To further identify key gene associated with immune responses, we conducted PPI analysis of the upregulated DEGs using a web-based tool (**Figure 2C**) and found that the interactions between proteins were enriched for those involved in antiviral responses. Next, we analyzed patient survival curves according to expression levels of antiviral genes, *ISG20* was identified as the only gene significantly associated with overall survival; increased ISG20 expression was correlated with favorable survival in patients with ovarian cancer in both the TCGA and GSE9891 datasets (**Figure 2D**). These results suggest that ISG20 is important for regulation of immune responses to ovarian cancer.

### 3.3 High ISG20 expression is associated with elevated CD8+ T cell infiltration and ISG20 overexpression enhances ovarian cancer immunogenicity

**Figure 3:**
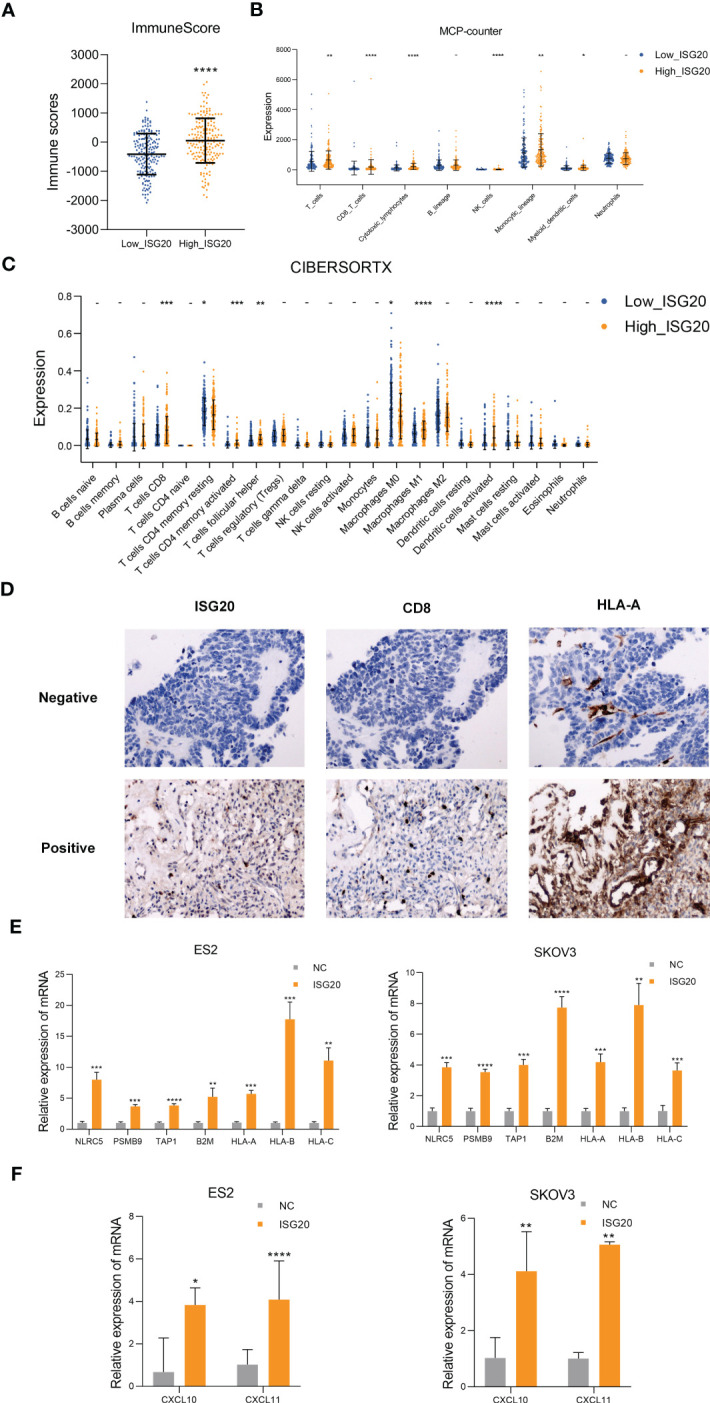
High *ISG20* expression is accompanied by increased CD8+ T cell infiltration in ovarian cancer, and cells overexpressing *ISG20* induce antigen presentation and chemokine production. **(A)** Comparison of ESTIMATE scores between the low and high *ISG20* groups (P < 0.05). **(B)** Comparisons of the abundances of 8 immune cell subpopulations between the low and high ISG20 groups based on MCP-counter analysis. **(C)** Comparison of proportions of 22 immune cell types between the low and high *ISG20* groups based on CIBERSORTX analysis. **(D)** Representative images of immunohistochemistry analyses of ISG20, CD8+, and HLA-A in ovarian cancer samples. **(E)** Real-time PCR analysis of levels of antigen presentation-associated genes in ES2 and SKOV3 cells overexpressing GFP and ISG20. **(F)** Real-time PCR analysis of chemokine gene expression levels in ES2 and SKOV3 cells overexpressing GFP and ISG20. Representative results of three independent experiments. The Mann-Whitney test was used for comparisons of tumor infiltrating immune cells. The unpaired two-tailed Student’s t-test was used for comparisons of real-time PCR results. *P < 0.05, **P < 0.01, ***P < 0.001 and ****P <0.0001.

To investigate the correlation between *ISG20* expression and immune cell infiltration, we compared the composition of the tumor microenvironment between the high and low *ISG20* subsets of TCGA ovarian cancer dataset. First, we used the ESTIMATE tool to infer all immune cells in TCGA ovarian cancer and concluded that the immune score was higher in the *ISG20* high subtype (**Figure 3A**). We then evaluated 8 kinds of immune cells between the two subtypes using MCP counter. In detail, patients with the high ISG20 subtype had significantly elevated percentages of T cells, CD8 T cells, cytotoxic lymphocytes, natural killer cells, monocytic lineage, and myeloid dendritic cells (**Figure 3B**). Moreover, we estimated the population abundance of tissue-infiltrating immune cells using CIBERSORTX, and the results were consistent with those generated using MCP counter (**Figure 3C**). Increased infiltration of CD8+ T cells was observed in paraffin-embedded ovarian cancer samples with high ISG20 expression, which was also consistent with TCGA RNA-seq analysis results (**Figure 3D**). These findings indicate that high ISG20 expression is associated with increased immune cell infiltration.

CD8+ T lymphocytes can eliminate tumor cells through T-cell receptor (TCR) recognition of tumor neoantigen peptides presented in the context of major histocompatibility complex class I (MHC-I) molecules. Malignancies with impaired antigen presentation caused by mutations, loss of heterozygosity, or reduced expression of MHC-I, are frequently resistant to immunotherapy (37, 38). Therefore, we detected antigen presentation-related genes in the ovarian cancer cell lines, ES2 and SKOV3, which overexpress ISG20. Real-time PCR analysis showed significantly increased expression of antigen presentation-related genes, including *NLRC5, PSMB9, TAP1, B2M, HLA-A, HLA-B, HLA-C* (**Figure 3E**). Increased *HLA-A* expression was also observed in paraffin-embedded ovarian cancer samples with high ISG20 expression (**Figure 3D**).

The CX2CL9, CXCL10, CXCL11/CXCR3 axis can inhibit tumors by participating in regulation of immune cell migration, differentiation, and activation (13, 39). DEGs in the high IFN signatures subtype showed upregulation of the chemokines, CXCL10 and CXCL11 (**Figure 2A**). Thus, we also examined CXCL9, CXCL10, CXCL11 in the ovarian cancer cell lines, ES2 and SKOV3, that overexpress ISG20, and found that CXCL10 and CXCL11 were significantly upregulated (**Figure 3F**). CXCL9 expression was not detected in ES2 and SKOV3 cells. CXCL9 is mainly induced by IFN-γ, while CXCL10 and CXCL11 are induced by IFN-α/β and IFN-γ (39); therefore, we inferred that ISG20 may upregulate CXCL10 and CXCL11 by inducing type I IFN.

### 3.4 Overexpression of ISG20 induces IFN-β production and STAT1 activation

**Figure 4:**
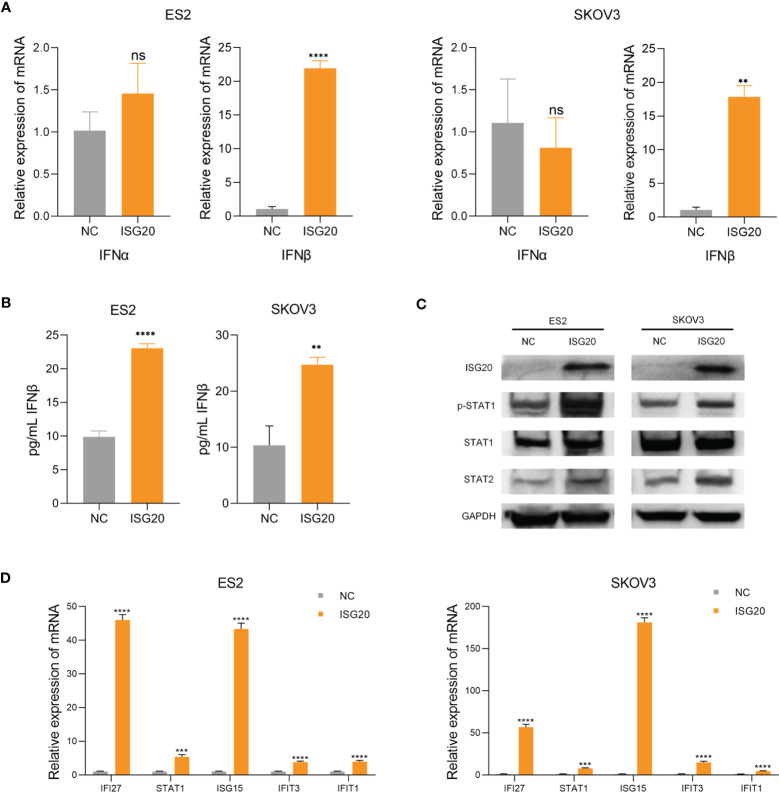
Overexpression of ISG20 induces IFN-β production and STAT1 activation. **(A)** Real-time PCR analysis of IFN-α and IFN-β gene expression levels in ES2 and SKOV3 cells overexpressing GFP and ISG20. **(B)** ELISA of IFN-β in culture media from ES2 and SKOV3 cells overexpressing GFP and ISG20. **(C)** Immunoblot analysis of ES2 and SKOV3 cells overexpressing GFP and ISG20. **(D)** Real-time PCR analysis of ISG expression in ES2 and SKOV3 cells overexpressing GFP and ISG20. Representative results of three independent experiments. **P < 0.01, ***P < 0.001 and ****P <0.0001 by unpaired two-tailed Student’s t-test. ns, not significant.

Based on our results, we hypothesized that ISG20 can induce type I IFN in ovarian cancer. To test this hypothesis, we overexpressed ISG20 in SKOV3 and ES2 ovarian cancer cells. The supernatants from ISG20 overexpressing cells contained higher concentration of type I IFN-β, while levels of IFN-α did not change in response to ISG20 overexpression (**Figures 4A, B**). Increased levels of p-STAT1, STAT1, and STAT2 were detected in SKOV3 and ES2 cells overexpressing ISG20 (**Figure 4C**). Further, an interferon response induced by IFN-β was also observed, in which a panel of ISGs (*IFI27, STAT1, IFIT1, IFIT3, ISG15*) was upregulated (**Figure 4D**).

### 3.5 ISG20 targets the RIG-I signaling axis leadings to IFN-β expression

**Figure 5:**
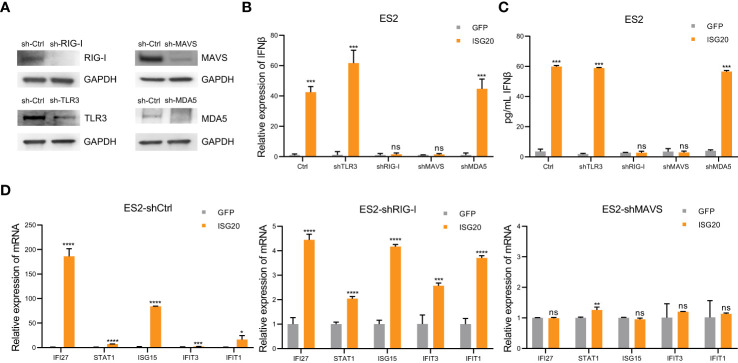
The interferon response to ISG20 overexpression is mediated by RIG-I. **(A)** Immunoblot analysis to validate knockdown of protein expression in ES2 cells transduced with the indicated shRNAs. **(B)**, **(C)** Real-time PCR analysis of IFN-β genes expression **(B)** and IFN-β levels in the supernatant **(C)** on ISG20 overexpression in ES2 cells with stable knockdown of TLR3, MDA5, RIG-I, or MAVS, and controls. **(D)** Expression levels of ISGs on ISG20 overexpression in ES2 cells with stable knockdown of RIG-I or MAVS and controls. Representative results of three independent experiments. *P < 0.05, **P < 0.01, ***P < 0.001 and ****P <0.0001 by unpaired two-tailed Student’s t-test. ns, not significant.

The engagement of cytoplasmic pattern recognition receptors in response to pathogen-associated molecular patterns (PAMPs) can induce interferon response, thus affecting the innate and adaptive immune systems (40). PAMPs, including lipopolysaccharide, dsRNA, double-stranded DNA, CpG DNA, and single-stranded RNA, can induce IFN-β production. Considering the function of ISG20 in degrading hepatitis B virus (HBV) epsilon RNA and spliceosomal U small nuclear RNA molecules (snRNAs), we hypothesized that endogenous dsRNA could function as a mediator between ISG20 and IFN-β (24, 41). Sensors of dsRNA mainly include MDA5, toll-like receptor 3 (TLR3), and RIG-I. Therefore, We constructed ES2 cells with TLR3, RIG-I, MDA5, and MAVS stably knocked down (**Figure 5A**). RIG-I knockdown almost abrogated IFN-β induction *via* ISG20 overexpression (**Figures 5B, C**). MAVS is an intermediary protein necessary in mediating IFN-β induction and MAVS knockdown also resulted in no IFN-β induction on ISG20 overexpression (**Figures 5B, C**). The ISGs induced by IFN-β were also almost abrogated in cells with RIG-I and MAVS knocked down cells on ISG20 overexpression (**Figure 5D**). Overall, these results indicate that on ISG20 overexpression, the RIG-I/MAVS signaling pathway participates in inducing the IFN-β production.

### 3.6 ISG20 cleaves dsRNA depending on its ribonuclease activity, leading to dsRNA accumulation

**Figure 6:**
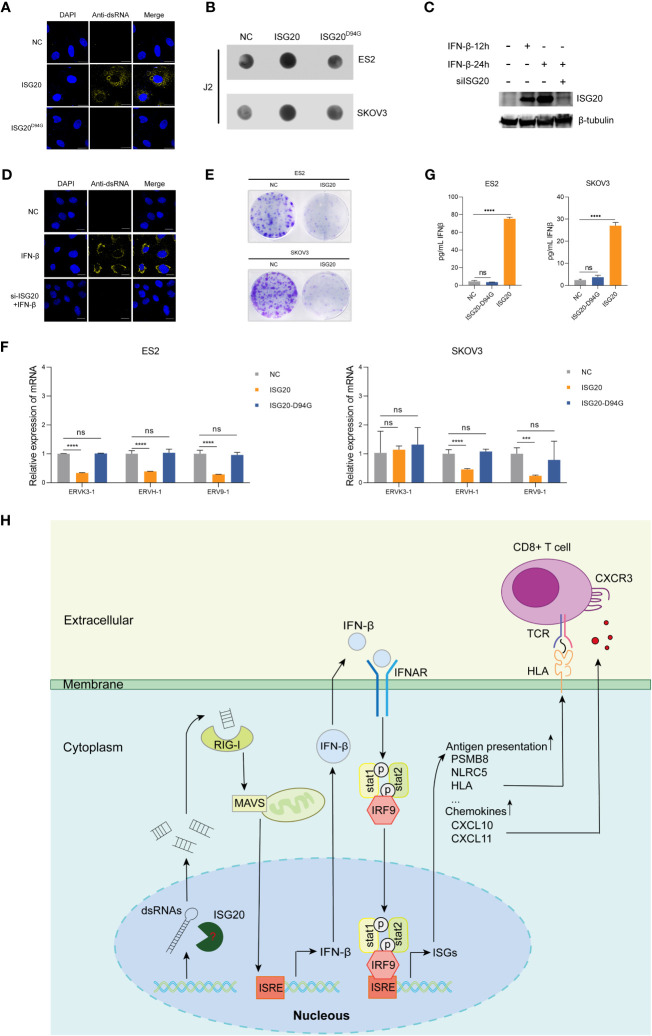
Overexpression of ISG20 triggers cytoplasmic accumulation of endogenous dsRNA, which depends on ISG20 ribonuclease activity. **(A)** Immunofluorescence staining analysis of endogenous dsRNA in SKOV3 cells overexpressing ISG20. Scale bars, 20μm. **(B)** Dot blot analysis of total RNAs samples extracted from ES2 and SKOV3 cells overexpressing GFP or ISG20. **(C)** Immunoblot analysis of ISG20 in SKOV3 cells in the presence of IFN-β with or without siISG20. **(D)** Immunofluorescence staining analysis of endogenous dsRNA in SKOV3 cells in the presence of IFN-β with or without siISG20. Scale bars, 20μm. **(E)** Colony formation assays in ES2 and SKOV3 cells overexpressing GFP or ISG20. **(F)** ELISA of IFN-β in the culture media from ES2 and SKOV3 cells overexpressing GFP, ISG20, and ISG20^D94G^. **(G)** Real-time PCR analysis of ERV genes in ES2 and SKOV3 cells overexpressing ISG20. **(H)** Diagram illustrating the mechanism of ISG20 induction of IFN-β production in ovarian cancer cells. Representative results of three independent experiments. ***P < 0.001 and ****P < 0.0001 by unpaired two-tailed Student’s t-test. ns, not significant.

ISG20 has strong exonuclease activity for single-stranded RNA and DNA (42). Moreover, ISG20 can also directly interact with the epsilon stem-loop structure of viral RNA to carry out its ribonuclease activity (24). Further, ISG20 can promote the degradation of nascent spliceosomal U snRNAs and U1 variants, RIG-I primarily recognizes dsRNA molecules < 300 bp (18). Therefore, we hypothesized that ISG20 may cleave endogenous long dsRNA into large amounts of small dsRNA fragments. To test this hypothesis, we examined the concentration of endogenous dsRNA in SKOV3 cells using an anti-dsRNA specific J2 antibody. According to our immunostaining analysis, ISG20 overexpression clearly increased the amount of endogenous dsRNA in SKOV3 cells (**Figure 6A**). Dot blot analysis also demonstrated increased levels of endogenous dsRNA in ES2 and SKOV3 cells overexpressing ISG20 (**Figure 6B**). ISG20 is a type I interferon-stimulated gene. We investigated whether the induction of ISG20 by IFN-β could lead to an increase in dsRNA in the presence or absence of siISG20 (**Figure 6C**). The results showed that IFN-β can upregulate the level of endogenous dsRNA in SKOV3 cells, and the phenomenon of IFN-β-induced increase in endogenous dsRNA disappeared when the expression of ISG20 induced by interferon β was suppressed (**Figures 6D**; **S1**). In previous studies, dsRNA accumulation led to cancer cell growth inhibition (15, 18). This phenomenon was also observed in cells overexpressing ISG20 (**Figure 6E**). Since ERVs are the main source of long dsRNA, we next detected ERV expression levels in ES2 and SKOV3 cells, and found that ES2 and SKOV3 cells overexpressing ISG20 exhibited decreased expression of ERVs (**Figure 6F**).

To investigate the involvement of ISG20 ribonuclease activity in IFN-β production, we constructed an ISG20^D94G^ mutant plasmid with ribonuclease activity abolished (42). Based on our results, ISG20^D94G^ overexpression in cells did not induce IFN-β production (**Figure 6G**), and endogenous dsRNA accumulation was not further intensified (**Figures 6A, B**). These results indicate that ISG20 mediates endogenous dsRNA decay, resulting in dsRNA accumulation and consequent activation of type I IFN responses (**Figure 6H**).

## 4 Discussion

In this study, we demonstrate that ISG20 plays an important role in activating the IFN signaling pathway in ovarian cancer cells. IFN induced by ISG20 causes upregulation of antigen presentation-related genes and chemokines, thus enhancing tumor immunogenicity and attracting T cell infiltration. The accumulation of endogenous dsRNA in ovarian cancer cells overexpressing ISG20 is sensed by RIG-I, leading to IFN-β production.

Previous studies have found that ISG20 overexpression is associated with IFN response in mouse embryonic fibroblasts and upregulation of IFN-β expression during pseudorabies virus infection (30, 43). While Wu et al. held that ISG20 could not induce IFN production in HEK293T cells. Here, we found that ISG20 can induce IFN-β production in ovarian cancer cells, suggesting that ISG20 overexpression induction of IFN-β production may depend on cell type, which warrants further investigation.

It remains unclear, how a nuclease can produce more dsRNA. Either this is a completely unknown, indirect mechanism leading to real accumulation of dsRNA or the nuclease activity somehow releases dsRNA that is accessible for the J2 antibody and/or RIG-I detection. Since the effect is much stronger after 30hrs than after 24hrs, it seems to be really an indirect, long-term effect. On the other hand, our findings suggest that the exonuclease activity of ISG20 contributes to the accumulation of dsRNA suggesting its potential role in degrading dsRNA into smaller fragments. These results are consistent with previous studies demonstrating that ISG20 can bind to epsilon RNA and U snRNAs, leading to their degradation *via* its exonuclease activity. Previous research has shown that various modifications to viral RNA, such as N6-methyladenosine modification of HBV RNA and 2’O-methylation modification of human immunodeficiency virus RNA, can affect the ability of the ISG20 protein to degrade viral RNA (26, 29). Therefore, the specific structure and modifications of endogenous dsRNA that can be degraded by ISG20 remains to be studied. Since ISG20 can localize in both the nucleus and cytoplasm (21), further research is also needed to investigate the specific cellular localization of ISG20 in degrading endogenous dsRNA (**Figure 6H**). These findings also imply that ISG20 may inhibit viral replication *via* degrading viral dsRNA.

Although the functions of most dsRNA remain ambiguous, there is evidence that dsRNA contributes to stimulation of antiviral responses, cell growth, and embryonic development. Recent studies have reported that dsRNA regulation is associated with DNA and histone methylation, alternative splicing of Alu-enriched introns, and RNA helicase activity. In this study, we discovered that ISG20 function in inducing short dsRNA accumulation, providing new insights into dsRNA regulation. Since dsRNA accumulation can trigger IFN activation, there have been many attempts to cure various malignancies by combining dsRNA activating compounds, such as histone deacetylase inhibitors or DNA methyltransferases inhibitors, with immune checkpoint inhibitors (44). These combinations appear to enhance responses to immune checkpoint therapy. Thus, the results of this study provide a new target for the development of immunotherapy strategies for ovarian cancer.

## Data availability statement

The original contributions presented in the study are included in the article/**Supplementary Material**. Further inquiries can be directed to the corresponding authors.

## Author contributions

ZC contributed to the conception, design of the study, and performed the statistical analysis. ZC, MY, and HJ performed the research. ZC wrote the first draft of the manuscript. All authors contributed to the article and approved the submitted version.
