# Supplementary figures and images for: ISG20 stimulates anti-tumor immunity via a double-stranded RNA-induced interferon response in ovarian cancer
Source: Front Immunol. 2023 Jun 5;14:1176103. doi: 10.3389/fimmu.2023.1176103 (PMC10277467; doi:10.3389/fimmu.2023.1176103)

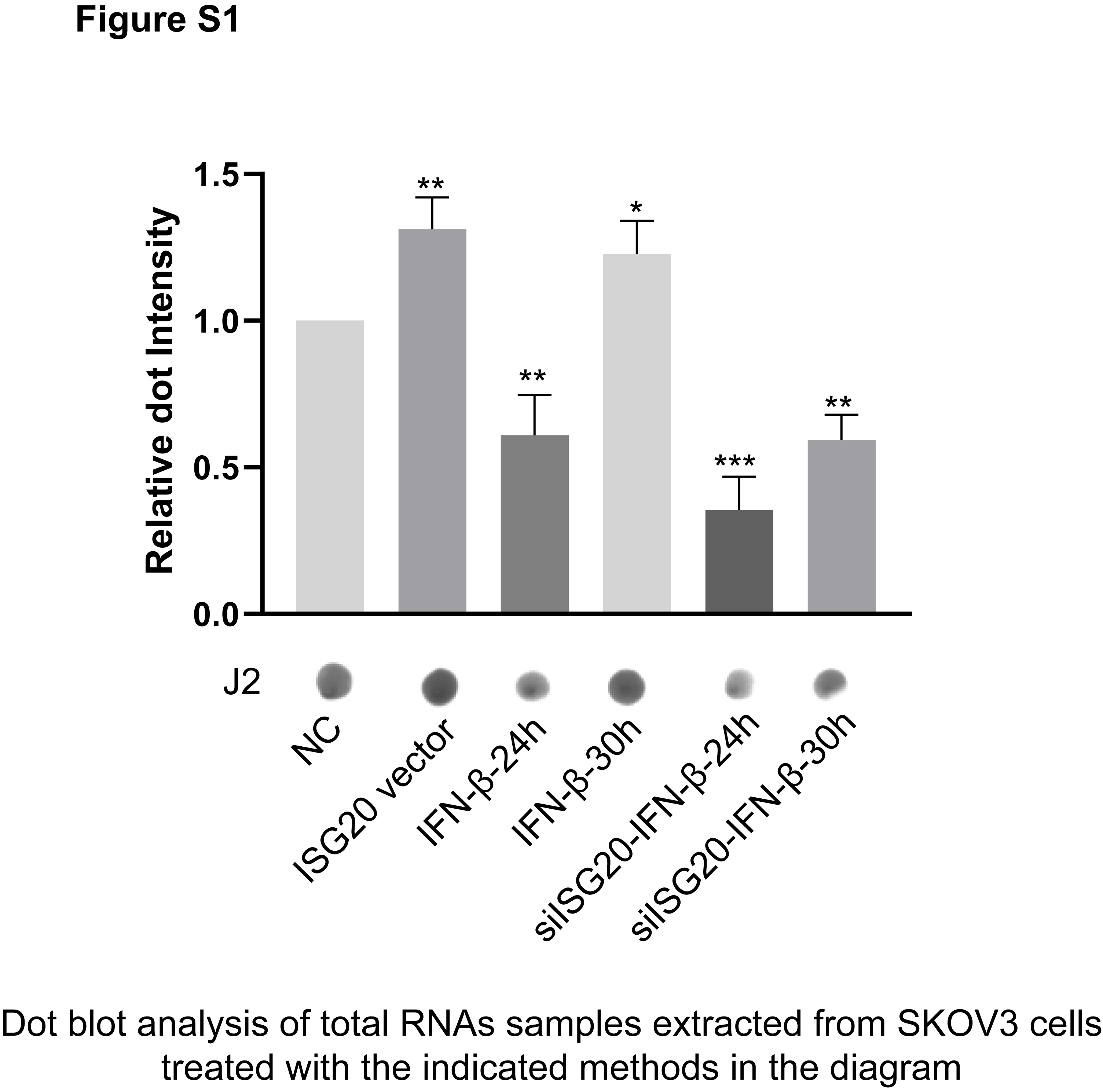

Supplement: Supplementary file 1 [file Image_1.tif]
